# Phosphorylation of EB1 regulates the recruitment of CLIP-170 and p150^glued^ to the plus ends of astral microtubules

**DOI:** 10.18632/oncotarget.14222

**Published:** 2016-12-25

**Authors:** Jie Ran, Youguang Luo, Yijun Zhang, Yang Yang, Miao Chen, Min Liu, Dengwen Li, Jun Zhou

**Affiliations:** ^1^ Institute of Biomedical Sciences, College of Life Sciences, Key Laboratory of Animal Resistance Biology of Shandong Province, Shandong Normal University, Jinan, Shandong 250014, China; ^2^ State Key Laboratory of Medicinal Chemical Biology, College of Life Sciences, Nankai University, Tianjin 300071, China

**Keywords:** microtubule, EB1, phosphorylation, CLIP-170, p150^glued^

## Abstract

Phosphorylation of end-binding protein 1 (EB1), a key member of microtubule plus end-tracking proteins (+TIPs), by apoptosis signal-regulating kinase 1 (ASK1) has been demonstrated to promote the stability of astral microtubules during mitosis by stimulating the binding of EB1 to microtubule plus ends. However, the roles of other members of the +TIPs family in ASK1/EB1-mediated regulation of astral microtubules are unknown. Herein, we show that ASK1-mediated phosphorylation of EB1 enhances the localization of cytoplasmic linker protein 170 (CLIP-170) and p150^glued^ to the plus ends of astral microtubules. Depletion of ASK1 or expression of phospho-deficient or phospho-mimetic EB1 mutants results in changes in the levels of plus-end localized CLIP-170 or p150^glued^. Mechanistic studies reveal that EB1 phosphorylation promotes its interactions with CLIP-170 and p150^glued^, thereby recruiting these +TIPs to microtubules. Structural analysis suggests that serine-40 is the primary phosphorylation site on EB1 that exerts these effects. Together, these findings provide novel insight into the molecular mechanisms that regulate the interactions of EB1 with other +TIPs.

## INTRODUCTION

During mitosis, accurate chromosome alignment requires dynamic attachment of sister kinetochores to spindle microtubules, in addition to the formation of interactions between astral microtubules and the cell cortex [1–3]. To satisfy the mitotic spindle checkpoint and facilitate the onset of anaphase, the kinetochores and spindle microtubules and their associated proteins must be precisely integrated and organized, while astral microtubules need to be stabilized. Defects in coordinating these events are associated with chromosome missegregation and genome aneuploidy, leading to cancer or developmental defects [1–3].

End-binding protein 1 (EB1), as a member of the microtubule plus end-tracking proteins (+TIPs), plays a central role in the regulation of microtubule dynamics by recruiting other +TIPs and binding partners to microtubule plus ends [4–6]. One such +TIP is cytoplasmic linker protein 170 (CLIP-170), which associates with EB1 and tubulin to dynamically track plus ends of growing microtubules and positively regulate microtubule growth [4, 7]. This function is dependent on high-affinity interactions between the CAP-Gly domain of CLIP-170 and the EEY motifs of EB1 and tubulin [7, 8]. During mitosis, CLIP-170 dynamically binds both unattached kinetochores and EB1 on microtubule tips to facilitate the kinetochore–microtubule attachment [9, 10]. CLIP-170 can also form a complex with other proteins that links microtubules to the cell cortex in order to respond to cell polarity cues [11].

p150^glued^, another +TIPs family member, is the largest subunit of the dynein/dynactin complex and has been shown to mediate cargo transport and promote microtubule stability [4]. Its plus end-tracking ability depends on both EB1 and CLIP-170 [12]. *In vitro* assays have demonstrated that p150^glued^ stabilizes microtubules by associating with EB1 [13]. Similarly, binding of EB1 to p150^glued^ is also required for astral microtubule elongation and cleavage furrow initiation during anaphase onset, underscoring the importance of the EB1-p150^glued^ interaction in connecting astral microtubules to the cell cortex [14].

An increasing number of studies have shown that the interplay between EB1 and other +TIPs is dependent on post-translational modifications of EB1 [15–21]. We have previously demonstrated the importance of ASK1-mediated phosphorylation of EB1 on its three serine/threonine residues for spindle orientation [17]. Importantly, the microtubule plus end-tracking ability of EB1 is enhanced by ASK1 phosphorylation [17]. However, the underlying mechanisms by which ASK1-mediated phosphorylation of EB1 contributes to astral microtubule stability remain elusive. In this study, we have further explored the relationship between ASK1 and EB1 by examining its effects on other +TIPs. Our results reveal that ASK1-dependent, EB1-mediated recruitment of CLIP-170 and p150^glued^ is essential for astral microtubule stability during mitosis.

## RESULTS

### ASK1-mediated phosphorylation of EB1 is essential for microtubule plus end tracking and astral microtubule stability

**Figure 1:**
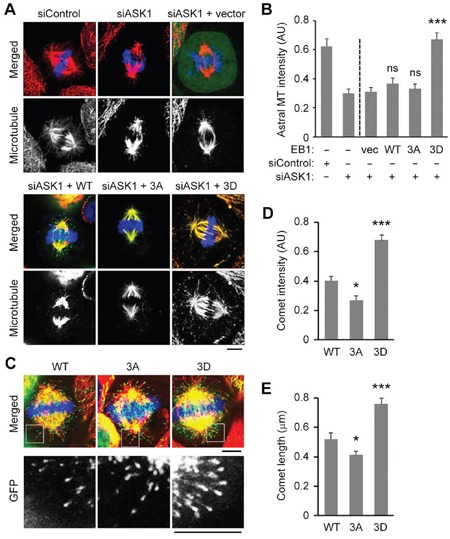
ASK1-mediated phosphorylation of EB1 is essential for astral microtubule stability and binding of EB1 to the plus ends of astral microtubules **A**. HeLa cells were transfected with control or ASK1 siRNAs, together with expression vectors carrying GFP-EB1 WT, 3A, or 3D. Cells were then stained with α-tubulin (red) antibodies and DAPI (blue), and metaphase cells were imaged. Scale bar, 5 μm. **B**. Experiments were performed as in (A), and astral microtubule intensity was quantified using Image J. n = 30 cells per group. **C**. HeLa cells were transfected with GFP-EB1 WT, 3A, or 3D expression vectors and stained with α-tubulin (red) antibodies and DAPI (blue). Scale bars, 5 μm. **D-E**. Experiments were performed as in (C), and the comet intensity (D) and comet length (E) were measured. n = 30 cells per group. AU indicates arbitrary unit. Values represent the mean ± SEM. *p < 0.05, ***p < 0.001; ns, not significant.

We have previously shown that overexpression of a triple phospho-mimetic EB1 mutant (S40D/T154D/T206D; 3D), but not wild-type (WT) EB1 or a phospho-deficient mutant (S40A/T154A/T206A; 3A), rescued the loss of astral microtubules in cells depleted of ASK1 and EB1 [17]. These results suggest that ASK1-mediated phosphorylation of EB1 is required for astral microtubule stability. To gain further insight into the underlying mechanism, we began by characterizing the extent to which the EB1 3D mutant could rescue astral microtubule loss in HeLa cells treated with control or ASK1 siRNAs in the absence of EB1 depletion. Weak astral microtubule staining was evident in ASK1 knockdown cells, and this decrease in astral microtubules was rescued by expression of EB1 3D, even in the presence of endogenous EB1 (Figure 1A, 1B). In addition, we found that the expression of EB1 3D enhanced the localization of EB1 at the plus ends of astral microtubules, despite the presence of endogenous EB1 (Figure 1C-1E).

### ASK1-mediated phosphorylation of EB1 enhances the localization of CLIP-170 to the plus ends of astral microtubules

**Figure 2:**
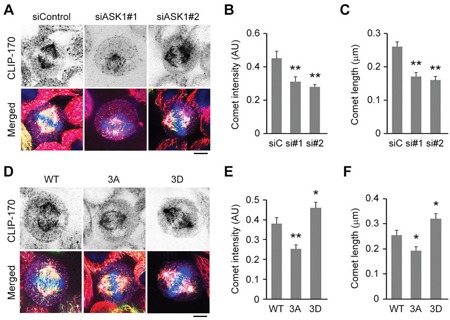
ASK1-mediated phosphorylation of EB1 enhances the localization of CLIP-170 to the plus ends of astral microtubules **A**. HeLa cells transfected with GFP-EB1 and control or ASK1 siRNAs were stained with α-tubulin (red) and CLIP-170 (violet) antibodies and DAPI (blue). Scale bar, 5 μm. **B-C**. Experiments were performed as in (A), and the CLIP-170 comet intensity (B) and comet length (C) were quantified. **D**. HeLa cells expressing GFP-EB1 WT, 3A, or 3D were stained with α-tubulin (red) and CLIP-170 (violet) antibodies and DAPI (blue). Scale bar, 5 μm. **E-F**. Experiments were performed as in (D), and the CLIP-170 comet intensity (E) and comet length (F) were quantified. AU indicates arbitrary unit. Values represent the mean ± SEM. *p < 0.05, **p < 0.01.

During mitosis, CLIP-170 dynamically localizes to unattached kinetochores to facilitate the establishment of kinetochore–microtubule attachments [11]. In addition to this localization pattern, we found that CLIP-170 also strongly localized to the plus ends of astral microtubules, suggesting that the interactions with CLIP-170 might regulate the dynamics of astral microtubules (Figure 2A). Therefore, we next sought to investigate whether ASK1-mediated phosphorylation of EB1 affected the localization of CLIP-170 to the plus ends of astral microtubules. Our results show that ASK1 knockdown in HeLa cells dramatically reduced the intensity and length of CLIP-170 comets at the plus ends of astral microtubules (Figure 2A-2C), indicative of a decrease in the binding of CLIP-170 to microtubule plus ends.

Similar results were observed in cells overexpressing the EB1 3A mutant (Figure 2D-2F). In contrast, overexpression of the EB1 3D mutant enhanced CLIP-170 localization at astral microtubule tips (Figure 2D-2F). Together, these results indicate that phosphorylation of EB1 by ASK1 promotes the localization of CLIP-170 to astral microtubule plus ends.

### ASK1-mediated phosphorylation of EB1 enhances the localization of p150^glued^ to the plus ends of astral microtubules

**Figure 3:**
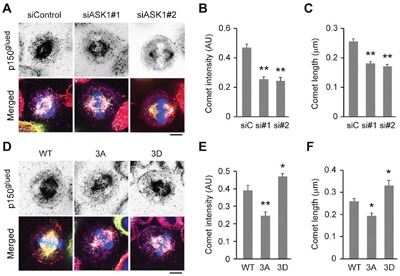
Phosphorylation of EB1 by ASK1 enhances the localization of p150^glued^ to the plus ends of astral microtubules **A**. HeLa cells transfected with GFP-EB1 and control or ASK1 siRNAs were stained with α-tubulin (red) and p150^glued^ (violet) antibodies and DAPI (blue). Scale bar, 5 μm. **B-C**. Experiments were performed as in (A), and the p150^glued^ comet intensity (B) and comet length (C) were quantified. **D**. HeLa cells transfected with GFP-EB1 WT, 3A, or 3D were stained with α-tubulin (red) and p150^glued^ (violet) antibodies and DAPI (blue). Scale bar, 5 μm. **E-F**. Experiments were performed as in (D), and the p150^glued^ comet intensity (E) and comet length (F) were quantified. AU indicates arbitrary unit. Values represent the mean ± SEM. *p < 0.05, **p < 0.01.

Like EB1 and CLIP-170, p150^glued^ localizes to the plus ends of astral microtubules, where it has been shown to play an essential role in connecting these microtubules to the cell cortex to facilitate chromosome alignment during mitosis. Therefore, to understand whether the localization of p150^glued^ is also regulated by ASK1-mediated phosphorylation of EB1, we tested whether its localization was affected by ASK1 depletion. Interestingly, treatment with ASK1 siRNAs dramatically reduced the intensity and length of p150^glued^ comets at the plus ends of astral microtubules (Figure 3A-3C).

Next, we examined whether overexpression of GFP-tagged EB1 mutants had a similar effect. In comparison to cells overexpressing WT EB1, cells overexpressing the phospho-deficient EB1 3A mutant exhibited decreased p150^glued^ comet intensity and length at astral microtubule tips, consistent with results from ASK1 depletion (Figure 3D-3F). Conversely, overexpression of the phospho-mimetic EB1 3D mutant exhibited significantly increased comet intensity and length (Figure 3D-3F). Together, these results suggest that phosphorylation of EB1 by ASK1 enhances the localization of p150^glued^ to the tips of astral microtubules, where it contributes to enhanced microtubule stability.

### Phosphorylation of EB1 by ASK1 promotes the binding of EB1 to CLIP-170 and p150^glued^

**Figure 4:**
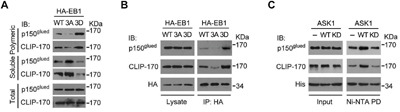
Phosphorylation of EB1 by ASK1 promotes its interaction with CLIP-170 and p150^glued^ **A**. Cells were transfected with expression vectors carrying HA-EB1 WT, 3A, or 3D and then washed in a buffer that prevents microtubule deploymerization. Polymeric and soluble tubulin fractions were then prepared. Levels of CLIP-170 and p150^glued^ present in the polymeric and soluble fractions were examined by immunoblotting. **B**. Cells were transfected with expression vectors carrying HA-EB1 WT, 3A, or 3D. HA-EB1 proteins were immunoprecipitated (IP) from cell lysates, and immunoprecipitates were analyzed for the presence of p150^glued^ and CLIP-170 by immunoblotting with the indicated antibodies. **C**. *In vitro* kinase assays were performed using ASK1 or ASK1 kinase dead mutant (KD) immunoprecipitated from cells, together with bacterially purified His-EB1. Phosphorylated His-EB1 was affinity isolated and then incubated with *in vitro* translated p150^glued^ or CLIP-170. Ni-NTA pulldown (PD) was then performed, and the precipitates were immunoblotted with CLIP-170 or p150^glued^ antibodies.

Because overexpression of the phospho-mimetic EB1 3D mutant enhanced the localization of CLIP-170 and p150^glued^ to the plus ends of astral microtubules, we sought to investigate whether this enhancement was due to direct recruitment by phosphorylated EB1. We first examined by cellular fractionation assays the interaction of CLIP-170 and p150^glued^ with microtubules in cells. Consistent with the decreased CLIP-170 and p150^glued^ comet intensities and lengths at the plus ends of astral microtubules in cells overexpressing EB1 3A, overexpression of EB1 3A in cells reduced the binding of CLIP-170 and p150^glued^ to microtubules (Figure 4A). In contrast, binding of both of these +TIPs to microtubules was enhanced by the overexpression of EB1 3D (Figure 4A), indicative of a positive role for phosphorylated EB1 in recruitment of CLIP-170 and p150^glued^ to microtubule tips.

Next, we examined the ability of EB1 or its mutants to immunoprecipitate CLIP-170 and p150^glued^ from cell lysates. The data revealed that the interaction between EB1 and CLIP-170 or p150^glued^ was enhanced by the phospho-mimetic 3D mutation, but was impaired by the phospho-deficient 3A mutation (Figure 4B).

To verify that ASK1 is the upstream kinase that regulates EB1-mediated recruitment of CLIP-170 and p150^glued^, we performed *in vitro* kinase assays with ASK1 to generate phosphorylated EB1, which was then incubated with *in vitro* translated CLIP-170 and p150^glued^. Nickel-nitrilotriacetic acid (Ni-NTA) pulldown assays revealed that, in comparison to controls lacking ASK1, incubation with WT ASK1 promoted binding of EB1 to CLIP-170 and p150^glued^, but incubation with an ASK1 kinase dead mutant did not. Together, these results indicate that the interaction of EB1 with CLIP-170 and p150^glued^ is enhanced by ASK1-mediated phosphorylation (Figure 4C).

### Structural analysis of the S40 and T206 mutants of EB1

**Figure 5:**
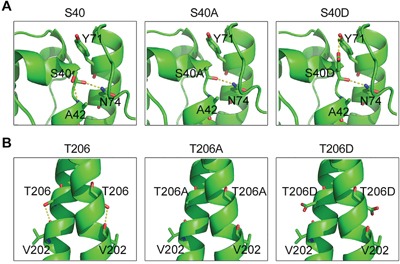
Structural analysis of the S40 and T206 mutants of EB1 **A**. In WT EB1, S40 forms stable hydrogen bonds with A42 and N74. Both the S40A and S40D mutations eliminate the hydrogen bond with A42, but S40D forms a new hydrogen bond with Y71. **B**. T206 forms a hydrogen bond with V202 in the WT protein, but both the T206A and T206D mutations eliminate this bond.

We next explored the structural basis for the effects of ASK1-mediated EB1 phosphorylation. The crystal structures of the EB1 N-terminus containing S40 (Protein Data Bank ID code: 1PA7) and the EB1 C-terminus containing T206 (Protein Data Bank ID code: 2H15) are available in the Protein Data Bank, but the structural data of the EB1 linker region containing T154 are not available. The S40 residue initially forms stable hydrogen bonds with A42 and N74 (Figure 5A). Both the S40A and S40D mutations eliminate the hydrogen bond with A42, but S40D forms a new hydrogen bond with Y71 (Figure 5A). Y71 and N74 are located in a helix that is almost parallel to the helix containing S40 (Figure 5A), suggesting that the S40-Y71 interaction may help to stabilize the EB1 N-terminus and contribute to its binding to microtubules and subsequent recruitment of other +TIPs. With respect to T206, this residue initially forms a hydrogen bond with V202. Although T206A and T206D do not appear to be involved in a direct interaction with CLIP-170 or p150^glued^ (Figure 5B), it is possible that the T206D mutation may make the linker region of EB1 more flexible, thereby promoting the binding of EB1 to microtubules and the recruitment of other +TIPs.

## DISCUSSION

The +TIPs family member EB1 plays essential roles in the regulation of microtubule dynamics during both interphase and mitosis by recruiting other +TIPs to microtubule plus ends. Our laboratory has previously demonstrated that ASK1 phosphorylates EB1 on S40, T154, and T206 [17]. We have also reported that ASK1-mediated phosphorylation of EB1 ensures accurate spindle orientation and positioning by enhancing the plus end-tracking ability of EB1 and promoting astral microtubule stability [17]. In this study, we continued our characterization of this mechanism by asking whether other +TIPs contribute to astral microtubule stability downstream of ASK1 and EB1. Results from this analysis revealed that the localization of CLIP170 and p150^glued^ to the tips of astral microtubules was enhanced by overexpression of EB1 3D. Furthermore, we found that ASK1-mediated phosphorylation of EB1 enhanced the interaction of EB1 with CLIP170 and p150^glued^.

Post-translational modifications of the EB1 family proteins have been an important field of study over the past decade [15–23]. We previously identified the proto-oncogene Src tyrosine kinase as an upstream regulator of EB1 that primarily phosphorylates Y247 to regulate cell migration [24]. Phosphorylation of EB1 by Src decreases its recruitment of adenomatous polyposis coli (APC) and mitotic centromere associated kinesin (MCAK) to microtubule plus ends, thereby affecting microtubule dynamics, which play a critical role in cell migration [24]. Phosphorylation of EB1 at S155 and T166 have an opposite effect on its accumulation at microtubule tips, implicating the liner domain of EB1 in its plus end-tracking ability [16]. In addition, tau-tubulin kinase 2, a newly identified +TIP, has been shown to regulate microtubule dynamics by associating with EB1 and another EB family member, EB3 [25].

Modification of EB1 has also been found to occur during mitosis. The budding yeast EB1 homolog, Bim1p, is phosphorylated by Ipl1 at its linker domain during anaphase, resulting in an almost total loss of the plus end-tracking ability of Bim1p [19]. However, only through release of Bim1p from spindle tips can timely disassembly and separation of the spindle midzone be ensured, since prior to anaphase, Bim1p plays essential roles in stabilizing the spindle and ensuring proper spindle elongation [19]. In mitotic cells, EB1 can be acetylated at K220 by p300/Creb-binding protein-associated factor (PCAF) if the connections between kinetochores and the spindle are not well established [18]. This modification of EB1 disrupts its interaction with APC and ensures that all chromosomes are aligned before the onset of anaphase [18]. The present study contributes to our understanding of the post-translational modifications of EB1 during mitosis by identifying a novel role for EB1 in regulation of astral microtubule stability during metaphase. Furthermore, our data indicate that ASK1/EB1-mediated recruitment of CLIP-170 and p150^glued^ to astral microtubule tips is required for this role.

The S40, T154, and T206 residues of EB1 were previously identified as targets of ASK1 phosphorylation. S40 is located in the calponin homology (CH) domain, which is responsible for the binding of EB1 to microtubule plus ends. Through structural analysis, we found that S40D formed a new hydrogen bond with Y71, but that S40 and S40A did not. This new connection between S40D and Y71 might stabilize the CH domain and contribute to microtubule plus end binding, since S40 and Y71 are located in two adjacent helices that form a nearly parallel bundle [13]. In contrast to S40, T154 and T206 are located in the flexible linker region and the linker region close to the EBH domain, respectively, neither of which is known to directly participate in microtubule binding or recruitment of CLIP-170 and p150^glued^ to microtubule plus ends. Nevertheless, an increasing number of reports demonstrate that the linker regions of EB1 play critical roles in regulating microtubule plus end binding [19, 23]. In addition to the residues mentioned above, PCAF-based super-resolution imaging has shown that several lysine residues in the EB1 linker region are important for tracking microtubule plus ends in live cells [26]. Therefore, the precise role of the linker region of EB1 in the regulation of microtubule dynamics needs further investigation.

At present, it remains elusive how EB1 phosphorylation at S40, T154, and T206 affects the interactions of EB1 with CLIP-170 and p150glued. The C-terminal EEY motif of EB1 is known to confer its interaction with CLIP-170 and p150glued by specifically recognizing the hydrophobic cavity of their CAP-Gly domains [7, 8]. Although the EEY motif of EB1 is not spatially close to S40, T154, or T206, it is possible that phosphorylation of these amino acids by ASK1 may induce conformational change that promotes the association of the EEY motif to the CAP-Gly domains.

**Figure 6:**
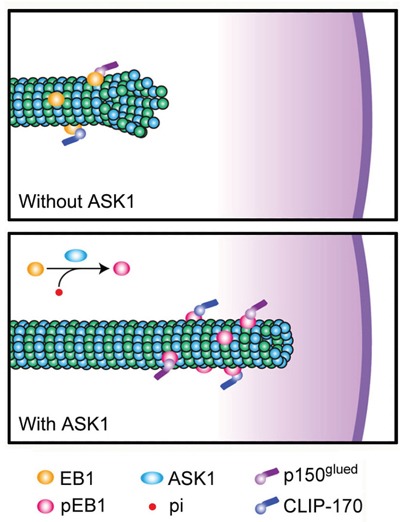
Proposed model for the function of ASK1-mediated phosphorylation of EB1 WT EB1 possesses a relatively weak ability to recruit CLIP170 and p150^glued^, leading to unstable astral microtubules. However, ASK1-mediated phosphorylation of EB1 promotes the recruitment of CLIP170 and p150^glued^ to the plus ends of astral microtubules through interactions with EB1, strengthening the connection between astral microtubules and the cell cortex.

Based on our findings, we propose a model to describe the relationship between ASK1-mediated phosphorylation of EB1 and the functions of CLIP170 and p150^glued^ (Figure 6). In this model, non-phosphorylated EB1 alone is not sufficient for the stabilization of astral microtubules, resulting in an increased likelihood of catastrophe and impaired chromosome alignment. ASK1-induced phosphorylation of EB1 not only increases its plus end-tracking ability, but also promotes its recruitment of CLIP170 and p150^glued^ to astral microtubules. These additional +TIPs further enhance astral microtubule stability and contribute to stable connections with the cell cortex during mitosis. While this study expands our knowledge of how microtubule dynamics are regulated during mitosis, further research is needed to provide a spatiotemporal map of the complex network of signaling proteins and interacting proteins that control microtubule functions and dynamics throughout the cell cycle.

## MATERIALS AND METHODS

### Antibodies and chemicals

Antibodies against HA and His (Sigma-Aldrich, St. Louis, MO, USA); CLIP-170 and p150glued (Santa Cruz Biotechnology, Santa Cruz, CA, USA); and ASK1 and α-tubulin (Abcam, Cambridge, MA, USA) were purchased from the indicated sources. Horseradish peroxidase-conjugated secondary antibodies were from Amersham Biosciences (Chandler, AZ, USA). Fluorescent dye-conjugated secondary antibodies were obtained from Jackson ImmunoResearch Laboratories (West Grove, PA, USA). DAPI was from Sigma-Aldrich.

### Plasmids, proteins and siRNAs

Mammalian expression plasmids for HA-EB1, GFP-EB1, ASK1, and various mutants were described previously [17, 27–29]. Purification of His-EB1 from bacteria and *in vitro* translation of ASK1 were described as previously [17]. Control siRNA (5′-CGUACGCGGAAUACUUCGA-3′) and ASK1 siRNAs (#1: 5′-GCACUCCUUCAUCGAGCU-3′; #2: 5′-GGUAUACAUGAGUGGAAUU-3′) were synthesized by Ribo Bio (Guangzhou, China).

### Cell culture and transfection

HeLa cells were purchased from the American Type Culture Collection (Manassas, VA, USA) and grown in Dulbecco's modified Eagle's medium supplemented with 10% fetal bovine serum at 37°C in 5% CO_2_. Plasmids were transfected into cells with polyethyleneimine (Sigma-Aldrich), and siRNAs were transfected with the lipofectamine RNAiMAX reagent (Invitrogen, Carlsbad, CA, USA).

### Fluorescence microscopy

Cells were fixed with methanol at -20°C for 5 min and blocked with 2% bovine serum albumin in phosphate-buffered saline. Cells were then incubated in succession with primary and secondary antibodies followed by staining with DAPI and examined with a TCS SP5 confocal microscope (Leica, Wetzlar, Germany) as described [30, 31]. The intensity and length of comets at astral microtubule plus ends were analyzed with the Image J software (National Institutes of Health, Bethesda, MD, USA) as described previously [32, 33].

### Immunoblotting, immunoprecipitation and Ni-NTA pulldown

Proteins were resolved by SDS/PAGE and transferred onto polyvinylidene difluoride membranes (Millipore, Billerica, MA, USA). The membranes were blocked and incubated with primary antibodies and then with horseradish peroxidase-conjugated secondary antibodies. Specific proteins were visualized with the enhanced chemiluminescence detection reagent (Millipore) as described previously [34]. For immunoprecipitation and Ni-NTA pulldown, cell lysates or protein mixtures were incubated with antibody- or Ni-NTA-coated agarose beads at 4°C for 2 h. The beads were washed and boiled in the SDS loading buffer, and the proteins were detected by immunoblotting.

### Preparation of polymeric and soluble tubulin fractions from cells

Cells were washed with phosphate-buffered saline, and soluble proteins were then extracted under conditions that prevent microtubule depolymerization (0.1% Triton X-100, 0.1 M MES, pH 6.75, 1 mM MgSO4, 2 mM EGTA, 4 M glycerol) as described previously [35]. The remaining polymeric tubulin fraction containing microtubules and associated proteins was dissolved in 0.5% SDS in 25 mM Tris (pH 6.8). Proteins present in the polymeric and soluble tubulin fractions were then analyzed by immunoblotting.

### Statistics

Analysis of statistical significance was performed by the Student's t-test for comparison between two groups and by the ANOVA test for multiple comparisons.
